# Endocardite infectieuse en milieu cardiologique Dakarois: étude descriptive à propos de 39 cas

**Published:** 2010-11-14

**Authors:** Mouhamadou Bamba Ndiaye, Maboury Diao, Adama Kane, Malick Bodian, Alassane Mbaye, Mouhamadoul Mounir Dia, Moustapha Sarr, Abdoul Kane, Serigne Abdou Ba

**Affiliations:** 1 Hôpital Aristide Le Dantec, Avenue Pasteur Dakar, Sénégal; 2 Hôpital Général Grand Yoff, Dakar, Sénégal

**Keywords:** Endocardite infectieuse, Cardiopathie rhumatismale, Dakar, Senegal

## Abstract

**Abstract:**

L’endocardite infectieuse est une complication fréquente des cardiopathies rhumatismales. L’objectif de ce travail était de faire une étude descriptive de l’endocardite infectieuse, en milieu hospitalier Dakarois.

Il s’agit d’une étude rétrospective, descriptive, réalisée à la clinique cardiologique de l’hôpital Aristide Le Dantec, durant la période allant de Janvier 2004 à Décembre 2008. Etaient inclus tous les patients hospitalisés et traités pour endocardite infectieuse certaine ou probable, selon les critères de Durack. Nous avons étudié les paramètres épidémiologiques, cliniques, biologiques et échocardiographiques.

Le nombre total d’admissions dans le service durant la période d’étude était de 3746 patients, dont 870 pour valvulopathies rhumatismales. Nous avions enregistré 39 cas d’endocardite infectieuse soit une prévalence de 1,04% et 4,48% valvulopathies rhumatismales. L’âge moyen de nos patients était de 24 plus ou moins 11,5 ans avec des extrêmes de 6 et 52 ans. Plus de la moitié des patients soit 58,9 % (23 patients) avaient moins de 25 ans. On notait une légère prédominance féminine avec un sex-ratio homes/femmes de 0,95. La porte d’entrée était essentiellement bucco-dentaire 40%. L’anémie était constante avec un taux d’hémoglobine moyen à 8,4g/dl. Les hémocultures étaient positives chez 6 patients et le *Staphylococcus Aureus* était le germe le plus retrouvé. L’électrocardiogramme avait montré des troubles du rythme et de la conduction respectivement dans 69,2 et 10,2% des cas. L’échographie cardiaque mettait en évidence des végétations chez tous les patients, une rupture de cordage dans 6 cas et un abcès chez trois patients.

L’endocardite infectieuse constitue encore une réalité dans nos régions. Elle survient habituellement sur cardiopathie rhumatismale. Son diagnostic repose sur les hémocultures et l’échocardiographie.

## Introduction

L’endocardite infectieuse est un état septicémique, que constitue la greffe d’un agent pathogène, sur un endocarde sain ou préalablement lésé ou sur une prothèse valvulaire. Cette définition englobe les infections développées sur les malformations cardiaques et sur les sondes de pacemaker [1].

Il s’agit d’une atteinte infectieuse de l’endocarde, responsable de lésions essentiellement valvulaires, avec un risque important d’insuffisance cardiaque et d’embolies septiques. La valve la plus fréquemment concernée est la valve mitrale dans 41 %, suivie de la valve aortique dans 38 % [2]. Les manifestations cliniques de l’endocardite sont multiples et diffèrent selon l’agent étiologique et les facteurs de risque sous-jacents, mais aucune n’est suffisamment spécifique et sensible pour permettre de poser le diagnostic [3].

L’objectif de notre travail était de décrire les aspects épidémiologiques, cliniques et para-cliniques des endocardites infectieuses en milieu cardiologique à Dakar.

## Méthodes

Il s’agit d’une étude rétrospective, transversale, descriptive réalisée à la clinique cardiologique de l’Hôpital Aristide Le Dantec (Dakar, Sénégal). Le travail a porté sur 39 dossiers de patients hospitalisés et traités pour endocardite infectieuse de Janvier 2004 à Décembre 2008. Soixante-sept (67) dossiers ont été dépouillés, 28 ont été exclus en tenant compte des critères d’inclusion et/ou de non inclusion. Etaient inclus tous les patients hospitalisés et traités pour endocardite infectieuse certaine ou probable, selon les critères de Durack de la Duke University [4]. N’étaient pas inclus dans l’étude, les patients hospitalisés pour suspicion d’endocardite infectieuse et les dossiers incomplets.

Sur le plan épidémiologique, nous avions apprécié: l’âge, le sexe, les antécédents de cardiopathies de chirurgies cardiaques et d’endocardites. Sur le plan clinique l’interrogatoire a recherché une fièvre, une dyspnée, des précordialgies, des palpitations, une toux et une altération de l’état général (asthénie, anorexie, amaigrissement).

Les constantes cliniques suivantes ont été appréciées à l’entrée: la tension artérielle, la fréquence cardiaque, la fréquence respiratoire, la température, le poids, la taille et l’indice de masse corporelle.

L’examen physique avait recherché des signes cardiaques (cardiopathie sous-jacente, souffle piaulant, modification souffle préexistant), des signes cutanés (nodules d’Osler, érythème de Janeway, hippocratisme digital, purpura), une splénomégalie, une atteinte pleuro-pulmonaire (pneumopathie, pleurésie, hémoptysie) et des signes neurologiques.

Le fond d’œil, à la recherche de tache de Roth, avait été réalisé. Nous avions recherché une porte d’entrée (stomatologique, ORL, cutanée, pulmonaire et urinaire).

A la biologie, étaient appréciés le taux d’hémoglobine, le nombre de globules blancs, la vitesse de sédimentation, la C-réactive protéine, la fibrinémie, la créatininémie, le taux des antistreptolysines O, l’existence ou non d’une hématurie microscopique, une protéinurie et des hémocultures.

L’électrocardiogramme avait recherché des troubles du rythme et de la conduction. La radiographie du thorax de face avait permis de calculer le rapport cardio-thoracique et d’apprécier le parenchyme pulmonaire et la plèvre. L’échographie transthoracique permettait de rechercher des végétations (nombre et taille), une destruction valvulaire (abcès, mutilation, perforation), une cardiopathie (fuite ou sténose).

Les données recueillies ont été saisies et analysées avec le logiciel Epi Info version 3.5.1. L’étude descriptive s’est faite par le calcul ou la détermination: 1) des paramètres de position (fréquence pour les variables catégorielles et moyenne pour les variables quantitatives, médiane et mode); 2) des paramètres de dispersion (écart type, variance et étendue).

## Résultats

Le nombre total des admissions dans le service durant la période de l’étude était de 3746 patients, dont 870 pour valvulopathies rhumatismales. Nous avons enregistré 39 cas d’endocardites infectieuses soit une prévalence de 1,04% et 4,48% de valvulopathies rhumatismales. L’âge moyen de nos patients était de 24 plus ou moins 11,5 ans (extrêmes de 6 et 52 ans). Plus de la moitié des patients soit 58,9 % (23 patients) avaient moins de 25 ans. On notait une légère prédominance féminine avec un sex-ratio (H/F) de 0,95. Les antécédents (tableau 1) étaient dominés par la cardiopathie rhumatismale, l’insuffisance cardiaque et l’endocardite infectieuse. A l’admission une dyspnée était retrouvée chez 37 patients (94,9 %) avec des proportions de 32,4 %, 35,1 % et 32,4 % pour respectivement les stades II, III et IV de la New York Heart Association.

Des précordialgies et des palpitations étaient notées respectivement dans 20,5 % et 38,4 % de cas. L’examen physique révélait un amaigrissement (28 patients), une asthénie (34 patients), une anorexie (21 patients), une dénutrition (17 patients) avec un indice de masse corporelle moyen à 12,7 Kg/m^2^ (extrêmes de 7,8 et 22,4 Kg/m2) et une tachycardie dans 66,6 % (26 cas). Tous les patients présentaient une fièvre avec une température supérieure ou égale à 38°C chez 74,3 % (29 patients) d’entre eux (extrêmes de 37,5 et 41,1°C).

Au plan cardiaque l’examen notait une sémiologie d’insuffisance mitrale chez 30 patients, de maladie mitrale chez 6 patients, d’insuffisance aortique dans 23 cas et d’insuffisance tricuspide chez 15 patients. Une défaillance cardiaque était retrouvée chez 36 patients avec des proportions et un souffle piaulant était dans 7 cas. On notait également une hémoptysie dans 3 cas (7,7 %), une pneumopathie chez 9 malades, une pleurésie droite dans 3 cas et un accident neurologique chez 11 patients (28,2 %).

Ailleurs on notait une splénomégalie dans 1 cas et trois (3) cas d’hippocratisme digital. Le fond d’œil réalisé chez 15 patients n’avait pas montré de tâches de Roth. Les portes d’entrées (table 2) étaient bucco-dentaire (10 cas), pulmonaire (8 cas) et ORL (2 cas d’otite purulente et 1 cas de rhinite purulente) cutanée (1 cas) et urinaire (2 cas).

A l’hémogramme l’anémie était constante avec le taux d’hémoglobine moyen à 8,4 g/dl (extrêmes de 4,1 et 11,1 g/dl) et l’hyperleucocytose chez 21 patients (53 %). Le bilan inflammatoire montrait une vitesse de sédimentation à la première heure accélérée dans 94,8 % des cas, une hyperfibrinémie dans 64,1 % des cas et une C-réactive protéine élevée chez 35 patients. Le taux d’antistreptolysine O était supérieur ou égal à 400 UI/ml chez 5 patients et 7 patients (17,9 %) avaient présenté une élévation de la créatinine. Une protéinurie était retrouvée chez 4 patients (10,2 %) et une hématurie microscopique dans 3 cas (7,6 %).

Les hémocultures étaient positives chez 6 patients à *Staphylococcus aureus* chez 2 patients, à *Streptococcus Pneumoniae* (1 patient), à Escherichia coli (1 cas), à *Serratia Marcescens* (1 cas) et à un diplocoque non identifié chez 1 patient. L’examen cyto-bactériologique des urines avait permis de confirmer une porte d’entrée urinaire en isolant le même germe que celui identifié par l’hémoculture (Escherichia Coli), chez 1 malade.

**Figure 1:**
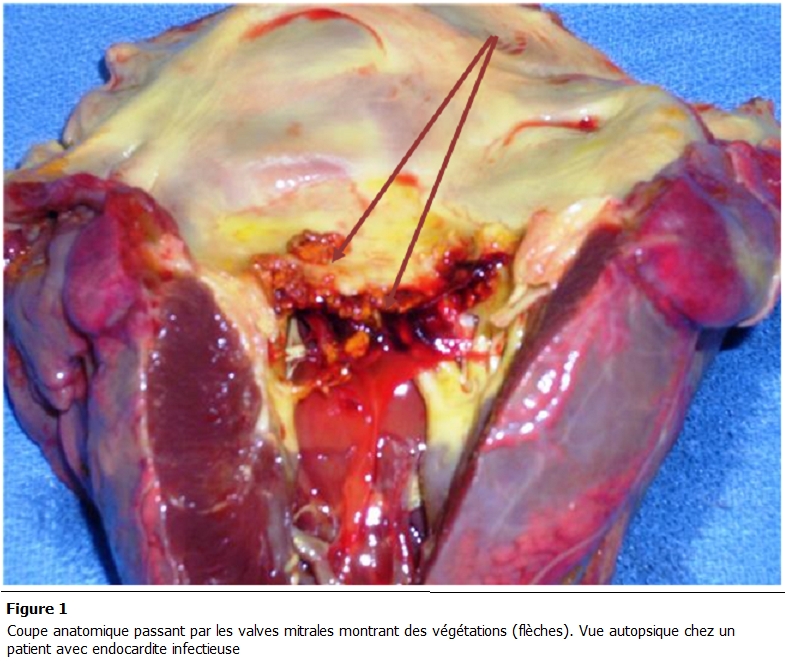
Coupe anatomique passant par les valves mitrales montrant des végétations (flèches). Vue autopsique chez un patient avec endocardite infectieuse

**Figure 3:**
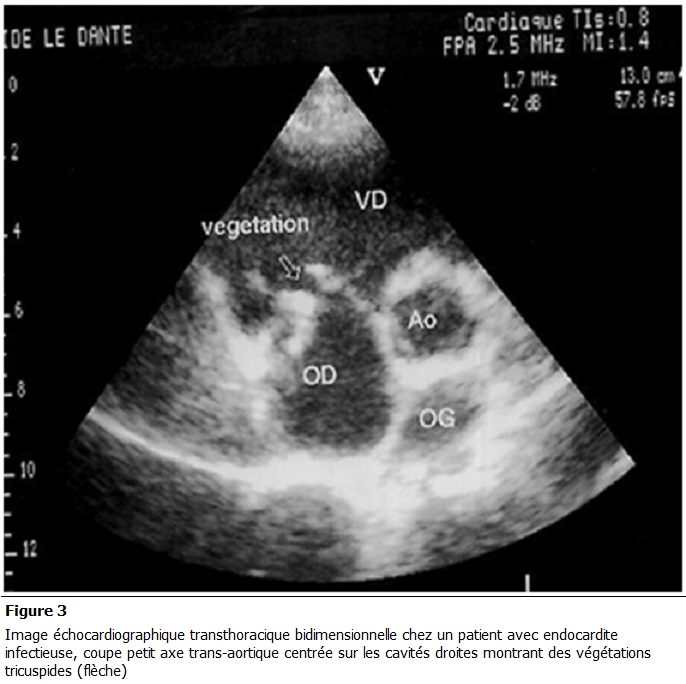
Image échocardiographique transthoracique bidimensionnelle chez un patient avec endocardite infectieuse, coupe petit axe transaortique centrée sur les cavités droites montrant des végétations tricuspides (flèche)

L’électrocardiogramme inscrivait 21 cas de tachycardie sinusale, 6 cas d’arythmie complète par fibrillation atriale et un trouble de la conduction intraventriculaire chez 4 patients. A la radiographie du thorax de face on notait une cardiomégalie dans 26 cas, une pleurésie droite chez trois patients et une pneumopathie dans 9 cas. L’échographie cardiaque mettait en évidence une ou plusieurs végétations chez tous les patients. Elles intéressaient le cœur gauche chez 38 patients (97,5 %) et le cœur droit dans 1 cas. Les végétations étaient situées au niveau mitral (figures 1 et 2) dans 66,6% (26 patients), aortique dans 20,5 % (8 patients), mitro-aortique dans 10,3 % (4 patients), tricuspidienne (figure 3) et pulmonaire chez un patient.

Les végétations étaient mobiles chez 15 patients et de grosse taille chez 13 patients. L’échographie cardiaque avait montré également 6 cas de rupture de cordage et 3 cas de prolapsus valvulaire. Elle avait par ailleurs mis en évidence respectivement un abcès de l’anneau mitral, un abcès de l’anneau aortique et un abcès septal, dans un cas. Un épanchement péricardique était retrouvé dans 2 cas (5,1 %). Au Doppler tous les patients présentaient au moins une fuite valvulaire: insuffisance mitrale dans 97,4 %, insuffisance aortique dans 59 %, insuffisance tricuspide dans 46,2 %. Une sténose valvulaire était retrouvée chez 8 patients (7 cas de sténose mitrale et 1 cas de sténose pulmonaire). Une hypertension artérielle pulmonaire importante à sévère était retrouvée dans 16 cas (41 %). Selon la classification de Durack, 26 patients avaient une endocardite possible et 13 une endocardite certaine. La durée moyenne d’hospitalisation était de 32,8 jours avec des extrêmes de 2 et 95 jours.

## Discussion

L’endocardite infectieuse est une maladie peu fréquente, mais restant grave, dont l’incidence semble être stable au cours des dernières décennies [5]. Elle touche la population jeune du fait de la prédominance, dans nos régions, des endocardites sur les valvulopathies rhumatismales. Ce constat est généralement noté dans la plupart des séries africaines [6-8].

La légère prédominance féminine, corrélée à l’importance des cardiopathies rhumatismales chez la femme, est parfois plus importante dans certains travaux africains [6-8]. Par contre, dans les séries occidentales, ce ratio a tendance à s’inverser en faveur des hommes (2 hommes pour une femme) [2]. L’importance de ces valvulopathies rhumatismales dans les antécédents confirme la persistance de ces affections en Afrique, contrairement aux nouvelles tendances concernant les endocardites sur pacemaker, prothèses valvulaires particulièrement [9,10].

Le pourcentage de porte d’entrée retrouvée dans notre étude est élevé par rapport à ceux observés dans la plupart des séries africaines [11]. Cependant il est inférieur à celui des séries occidentales [12]. Par contre dans environ 20% des cas aucune porte d’entrée n’est mise en évidence [12].

La prédominance habituelle de la porte d’entrée bucco-dentaire est également une constante dans notre étude comme dans beaucoup d’autres études [3,12-14].

La porte d’entrée pulmonaire vient en deuxième position, par contre elle reste rarement décrite dans la littérature africaine [7,11]. Les autres portes d’entrée présumées sont ORL, urinaire et cutanée. En ce qui concerne l’état cardiaque antérieur, l’endocardite infectieuse survient préférentiellement, sur un cœur préalablement lésé. En effet la prévalence de valvulopathie acquise varie entre 70% et 93,4% [5]. Ce constat est également lié à l’endémie rhumatismale dans nos régions. Sur le plan clinique la fièvre est un symptôme important de l’endocardite infectieuse et toute fièvre inexpliquée pendant plus de 10 jours chez un patient ayant un souffle cardiaque doit faire évoquer le diagnostic d’endocardite infectieuse [15].

L’amaigrissement, l’asthénie et l’anorexie sont également fréquents alors que la splénomégalie est rarement retrouvée [16]. Il en est de même pour les signes cutanéo-phanériens dans les séries africaines [5]. A la biologique, les auteurs s’accordent sur la grande fréquence de l’anémie et du syndrome inflammatoire biologique [7].

Le taux des endocardites infectieuses à hémocultures négatives était élevé dans notre série comme dans la littérature en Afrique [7,17]. Par contre ces endocardites infectieuses à hémocultures négatives sont relativement rares dans les pays occidentaux dans 5 à 10 % de cas [15,18-20]. En ce qui concerne les hémocultures positives, le germe le plus rencontré était le *Staphylococcus Aureus*, comme ce fut le cas pour certains auteurs [7].

L’échographie transthoracique reste un outil diagnostique indispensable dans nos régions [8] même si sa sensibilité et sa spécificité sont moindre que celle de l’échographie trans-œsophagienne [21].

## Conclusion

L’endocardite infectieuse constitue une réalité encore dans nos régions du fait de l’endémie des cardiopathies rhumatismale. Elle touche particulièrement les sujets jeunes de sexe féminin. Son diagnostic repose essentiellement sur les hémocultures et l’échographie cardiaque. La prévention de cette affection rejoint alors celle des valvulopathies rhumatismales qui repose sur la prise en charge des angines chez l’enfant.

## Conflits d’intérêts

Les auteurs ne déclarent aucun conflit d’intérêts

## Figures and Tables

**Table 1:** Principaux antécédents retrouvés dans une population de patients hospitalisés et traités pour endocardite infectieuse de Janvier 2004 à Décembre 2008 à la clinique cardiologique de l’Hôpital Aristide Le Dantec (Dakar, Sénégal)

**Antécédents**	**Nombre**	**Pourcentage (%)**
Cardiopathie rhumatismale	19	48,7
Insuffisance cardiaque	4	10,3
Endocardite infectieuse	3	7,6
Chirurgie (plastie aortique)	1	2,5
Cardiopathie congénitale	1	2,5

**Table 2:** Portes d’entrées d’endocardite dans une population de patients hospitalisés et traités pour endocardite infectieuse de Janvier 2004 à Décembre 2008 à la clinique cardiologique de l’Hôpital Aristide Le Dantec (Dakar, Sénégal)

**Porte d’entrée**	**Nombre**	**Pourcentage (%)**
Bucco-dentaire	10	41,6
Pulmonaire	8	33,3
ORL	3	12,5
Urinaire	1	8,3
Cutanée	1	4,1

**Figure 2:**
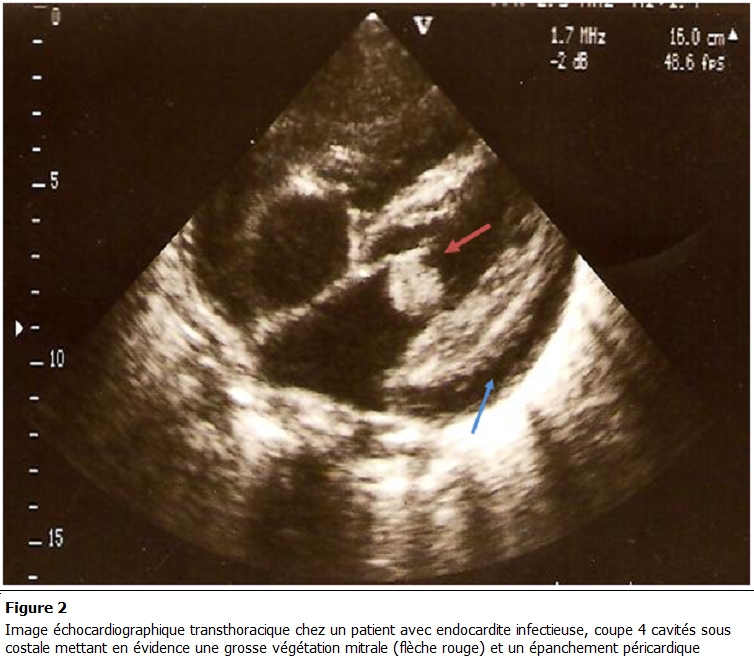
Image échocardiographique transthoracique chez un patient avec endocardite infectieuse, coupe 4 cavités sous costale mettant en évidence une grosse végétation mitrale (flèche rouge) et un épanchement péricardique
